# Transient use of hemolymph for hydraulic wing expansion in cicadas

**DOI:** 10.1038/s41598-023-32533-4

**Published:** 2023-04-18

**Authors:** Mary K. Salcedo, Tyler E. Ellis, Ángela S. Sáenz, Joyce Lu, Terrell Worrell, Michael L. Madigan, John J. Socha

**Affiliations:** 1 Biological and Environmental Engineering, Cornell University, Ithaca, NY USA; 2 Biomedical Engineering and Mechanics, Virginia Tech, Blacksburg, VA USA; 3 Entomology, University of Maryland, College Park, MD USA; 4 Industrial and Systems Engineering, Virginia Tech, Blacksburg, VA USA

**Keywords:** Biological techniques, Ecology

## Abstract

Insect wings must be flexible, light, and strong to allow dynamic behaviors such as flying, mating, and feeding. When winged insects eclose into adults, their wings unfold, actuated hydraulically by hemolymph. Flowing hemolymph in the wing is necessary for functioning and healthy wings, both as the wing forms and as an adult. Because this process recruits the circulatory system, we asked, how much hemolymph is pumped into wings, and what happens to the hemolymph afterwards? Using Brood X cicadas (*Magicicada septendecim*), we collected 200 cicada nymphs, observing wing transformation over 2 h. Using dissection, weighing, and imaging of wings at set time intervals, we found that within 40 min after emergence, wing pads morphed into adult wings and total wing mass increased to ~ 16% of body mass. Thus, a significant amount of hemolymph is diverted from body to wings to effectuate expansion. After full expansion, in the ~ 80 min after, the mass of the wings decreased precipitously. In fact, the final adult wing is *lighter* than the initial folded wing pad, a surprising result. These results demonstrate that cicadas not only pump hemolymph into the wings, they then pump it out, producing a strong yet lightweight wing.

## Introduction

When a winged insect transforms from a juvenile into an adult, its wings unfold and expand in an origami-like fashion, actuated hydraulically with hemolymph (insect blood)^1,2^. This pivotal process, which ultimately determines whether or not an insect can fly, recruits the insect’s circulatory system, switching temporarily from a system that transports nutrients and cells to a mechanical system that hydraulically and irreversibly expands the wings. Wing expansion is commonly investigated through the lens of genetic and hormonal pathways; how the wing changes *structurally* in this phase is less well understood^2–7^, especially for non-model/laboratory-raised insects.

Wing expansion occurs during adult insect ecdysis. It requires coordinated pumping from thoracic wing hearts (a type of accessory pulsatile organ)^8^ and the long, tubular dorsal vessel (which includes the main heart) to pressurize the thorax, drive hemolymph into the wing, and in some poorly understood way, pump out said hemolymph^2,6,9^. In fully formed adult wings, thoracic wing hearts are known to aspirate hemolymph from the wing, driving a current of hemolymph in either a circuitous or oscillating tidal pattern in the wing veins^1,2,10–12^. An insect can have multiple accessory pulsatile organs (e.g., antennal pulsatile organs); thoracic wing hearts can be separated from the main dorsal vessel or attached to the dorsal vessel in different configurations^8,9^. A recent study in mosquitoes used fluorescence to examine wing circulation and how an “unattached” thoracic wing heart pulls hemolymph from the wing 5× faster than the velocity at which it enters^10^. Investigating hemolymph circulation in the wing and the associated pumping organs which drive the flow requires static, laboratory experiments that are non-trivial in complexity^11,13^. In fact, slight disturbances can cause an insect to reverse its dorsal vessel pulsations^14^. Previous ecdysis studies in locusts and crickets focused on the various motor programs which drive flight muscle contraction and abdominal ventilation^15,16^ while simultaneously developing the pressure (typically measured in the gut) to initiate eclosion^9,13,17^, but wing expansion and the role of hemolymph was relatively ignored. Hemolymph and its movement, almost a third party to how it moves and where, has yet to be explored during a dynamic behavior such as wing expansion in insects.

Although adult insect ecdysis has well-stereotyped phases^17,18^, there is great variability between insects, especially non-laboratory species. In the field, the occurrence of cicada ecdysis in broods allows scientists to measure how “wild” insects deal with environmental fluctuations, and how often insects perform successful ecdysis^5,19^. The presence of cicadas in an ecosystem is critical, as they move a significant flux of nutrients as nymphs, their seasonal presence is a strong indicator of climate change on local biodiversity^20^, and they are influencers of geochemical forest cycles^21^. However, despite cicadas being a key ecological species^20^, we know little of their ecdysis or wing expansion dynamics. Thus we turned our focus towards a large brood of periodical cicadas to investigate wing expansion, and specifically the role of hemolymph during ecdysis, a previously unquantified phenomenon. We asked, how much hemolymph is pumped into the wing, and what happens to the hemolymph once expansion is complete?

## Results

**Figure 1:**
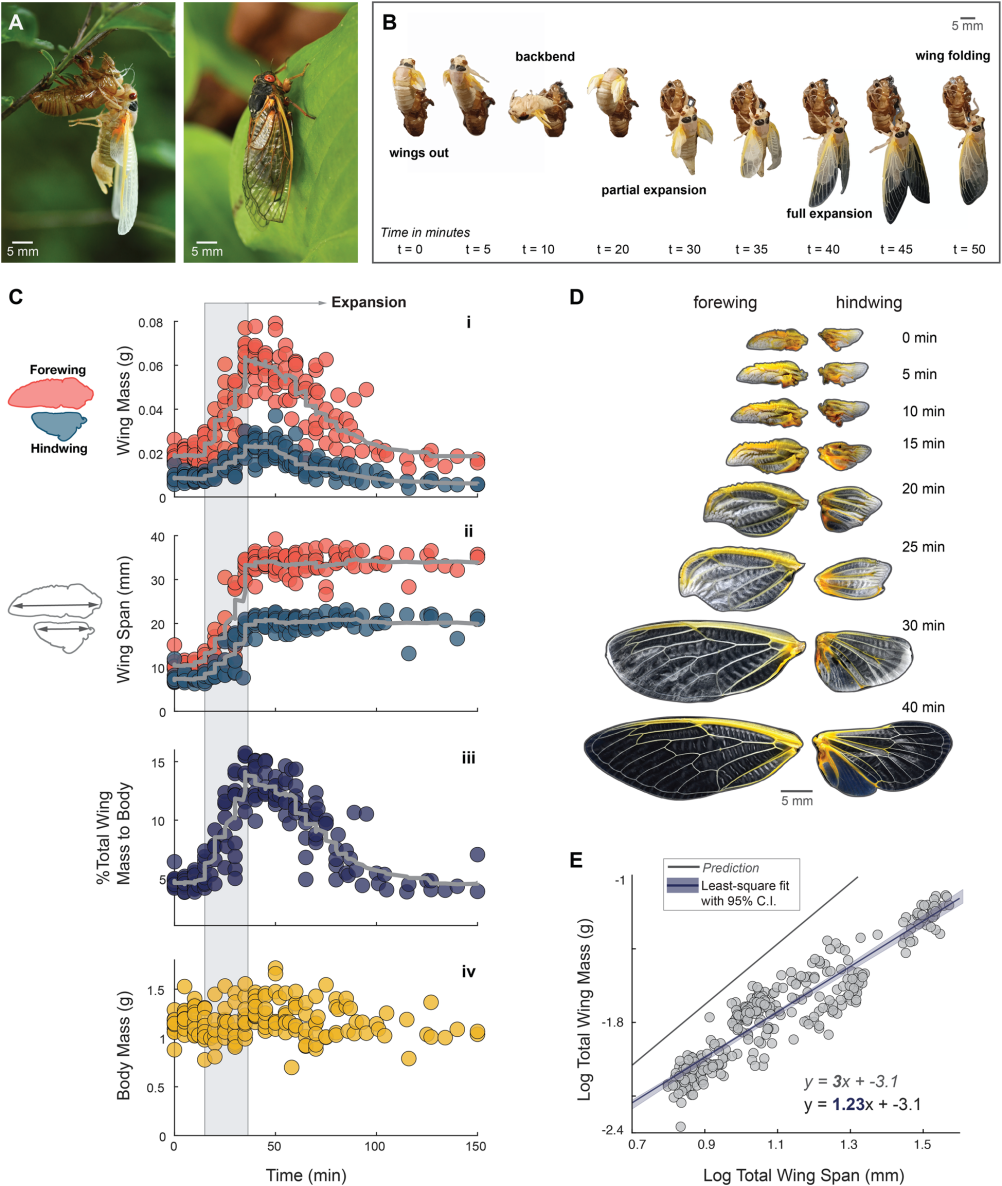
Wing expansion in cicadas. (**A**) Prior to wing expansion, juvenile cicada nymphs crawl up a vertical surface, quiesce, and initiate eclosion, the process of adult emergence (left). Sclerotization and melanization occur over the next 24 h (right). (**B**) To pull out its wings, *Magicicada septendecim* split the exoskeleton of the thorax and wriggle out. Four wings are freed during this process as the cicada continues to bend backwards. At about 40 min, the wings are fully expanded, and at 50 min they are folded over the abdomen. (**C**) Approximately 800 wings were dissected, weighed, and imaged in 5-min intervals during expansion from wings out (time t = 0 to 120 min). (**i**) Mass was measured immediately after dissection. Wing mass increased up until 40 min, then mass decreased over the next 2 h. (**ii**) Span (i.e., wing length) plateaued at 40 min, measured in FIJI post-experiment (See Fig. 3 for chord and area). (**iii**) At the end of the expansion phase, total wing mass accounts for ~16% of the insect’s mass (relative to body mass). (**iv**) Body mass over expansion. (**D**) Images of dissected fore- and hind-wings throughout expansion. (**E**) Log of the total wing mass versus log of span (**i**–**iii**) compared predicted isometric versus actual scaling relationships, with a least-squares regression fit and a 95% confidence interval (See Fig. 3 for log of chord and area relationships).

**Figure 2:**
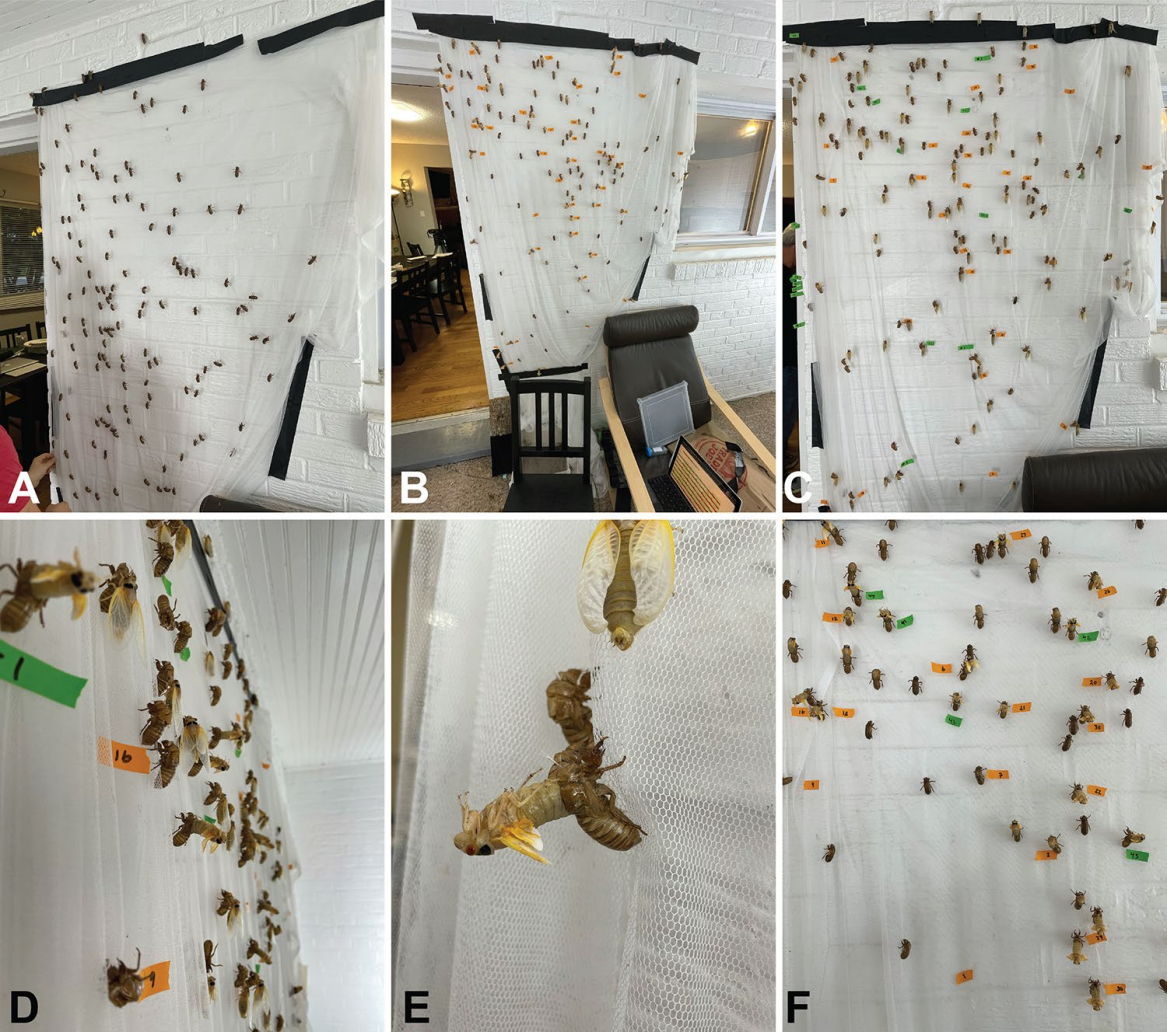
Eclosion and wing expansion on a “cicada wall”. An 8-foot wall of insect netting was mounted in a walled-in porch. Nymphs were placed on the wall and allowed to crawl freely until they settled to initiate emergence. (**A**) Nymphs have climbed up the mesh wall. (**B**) Cicadas emerging on the wall with tape labels indicating timing intervals. (**C**) As more cicadas emerge, more colors of tape are used to differentiate expansion at different times. (**D**) Side-view of the emergence wall. (**E**) A cicada in the backwards-bend phase of eclosion. When it flips forward to extract the abdomen, this is time "zero". (**F**) Due to the quick pace of emergence, many cicadas were not labelled. Cicadas then eclosed and were returned to their site of capture.

We investigated wing expansion in 17-year periodical Brood X cicadas (*Magicicada septendecim*, Fig. 1), taking advantage of a uniquely abundant and ephemeral ecological event (Fig. 1). In 2021, Brood X cicadas emerged in the millions after 17 years underground (densities up to 1000 cicadas/m^2^)^22^, which allowed for a higher sampling rate when compared with annual cicada species. We collected a total of 200 cicada nymphs and observed their transformation to adults (Fig. 1). Approximately 800 wings were dissected, weighed, and imaged in 5-min intervals over the course of 2 h (Fig. 2). Within 40 min, the neatly folded wing pads morphed into fully-formed adult wings; wing mass increased rapidly, reflecting that hemolymph was pumped into the wings. Impressively, forewing and hindwing mass increased nearly six times their initial wing mass (Fig. 1C,i,ii). This large increase in mass indicated that a substantial amount of hemolymph is shunted from the body to the wings, representing ~16% of the cicada’s total body mass (Fig. 1C,iii). Just after peak expansion, the mass of the wings decreased precipitously, resulting in final adult wings that were slightly *lighter* than the initial wing pads. These results demonstrate that cicadas not only transport hemolymph into the wings, they then transport it out, producing a fully-expanded, lightweight wing.

When a cicada nymph is ready to emerge, it dramatically changes lifestyle, going from an underground habitat where it feeds on xylem sap from roots of grasses or woody plants, to aerial as they seek to chorus, feed, and mate^14^. Cicadas pass through five nymphal instars, and when their final instar is ready, it makes its way to the surface^21^. To do this, a nymph digs out of the ground, finds a suitable perch, climbs up, and ecloses. Some terrestrial pupae can take minutes to hours to dig out to find a suitable substrate for eclosion^5,6,13^. Typically performed after dusk to avoid predators, emergence is cued by a suite of hormonal factors that cause the adult exoskeleton to soften within the nymph^5,18^. A layer of fluid is secreted between the old and new forms, the thoracic wing hearts and dorsal vessel work to pressurize the thorax with accumulating hemolymph^13^, and the backside of the nymph splits (see Supplementary Video), allowing the adult to emerge dorsally^2^. As the cicada wriggles its way out of its prior life stage, freeing four small and folded wings in a motion like pulling a sword from its sheath (Fig. 1B), it bends backwards (Fig. 1B), gulping air, a common strategy for hemimetabolous insects^16^. This air fills its digestive system, expanding it outwards as it pushes on the soft, but quickly hardening, exoskeleton. During this backwards movement, old tracheal linings (white threads) can be seen being pulled out of the adult, still attached to the nymph (now exuvia) (see Supplementary Video).

At this point a cicada’s wings are still tightly folded, but are free from the wing pad casing on the exuvia. For cicadas (and other hemimetabolous insects), wings develop in dorsal wing-pad casings^18^. Upon finishing gulping air (~few min) and allowing gravity to gently pull its long, extended abdomen out of the short nymph casing, the cicada snaps forward. This maneuver pulls the abdominal tip out of the casing, and the cicada is finally free and can potentially crawl to a more suitable location where subsequent wing expansion occurs. It begins expanding its wings, vigorously pumping its flight muscles to push hemolymph into the wing, as thoracic wing hearts pull fluid from the wing^13^. A developing wing is formed by two layers; during wing expansion and maturation, they seal together^2^. The wing grows in size; as it unfolds, and as pumping continues, its appearance changes from a cloudy opaque to clear (see Supplementary Video). Melanization and sclerotization will occur over the next 24–48 h, and normal coloration, such as spots or stripes, will appear^23^. Coloration may take up to 4–6 days to finalize depending on the insect species, and can be linked with achieving sexual maturity^24^.

To measure the mass and structural changes of wings during expansion, we set up a watch-and-wait experiment followed by rapid wing dissection and imaging (Fig. 1C,i–iv). Cicada nymphs were hand-collected between 4:30 and 7:00 pm EDT on May 15–19, 2021, and allowed to free-climb a mesh-covered wall (Fig. 2). Once an individual began molting, we designated “time zero” when each folded wing emerged from the wing pad (Fig. 1B). A total of 200 nymphs were sacrificed at 5-min intervals up to 120 min after time zero. Wings were dissected and weighed immediately, followed by imaging using a DSLR camera (Fig. 1C,i). Wing span (i.e., maximum length at each time point), chord (i.e., mid-span width at each time point), and area (Fig. 1C, ii and 3C) were measured.

**Figure 3:**
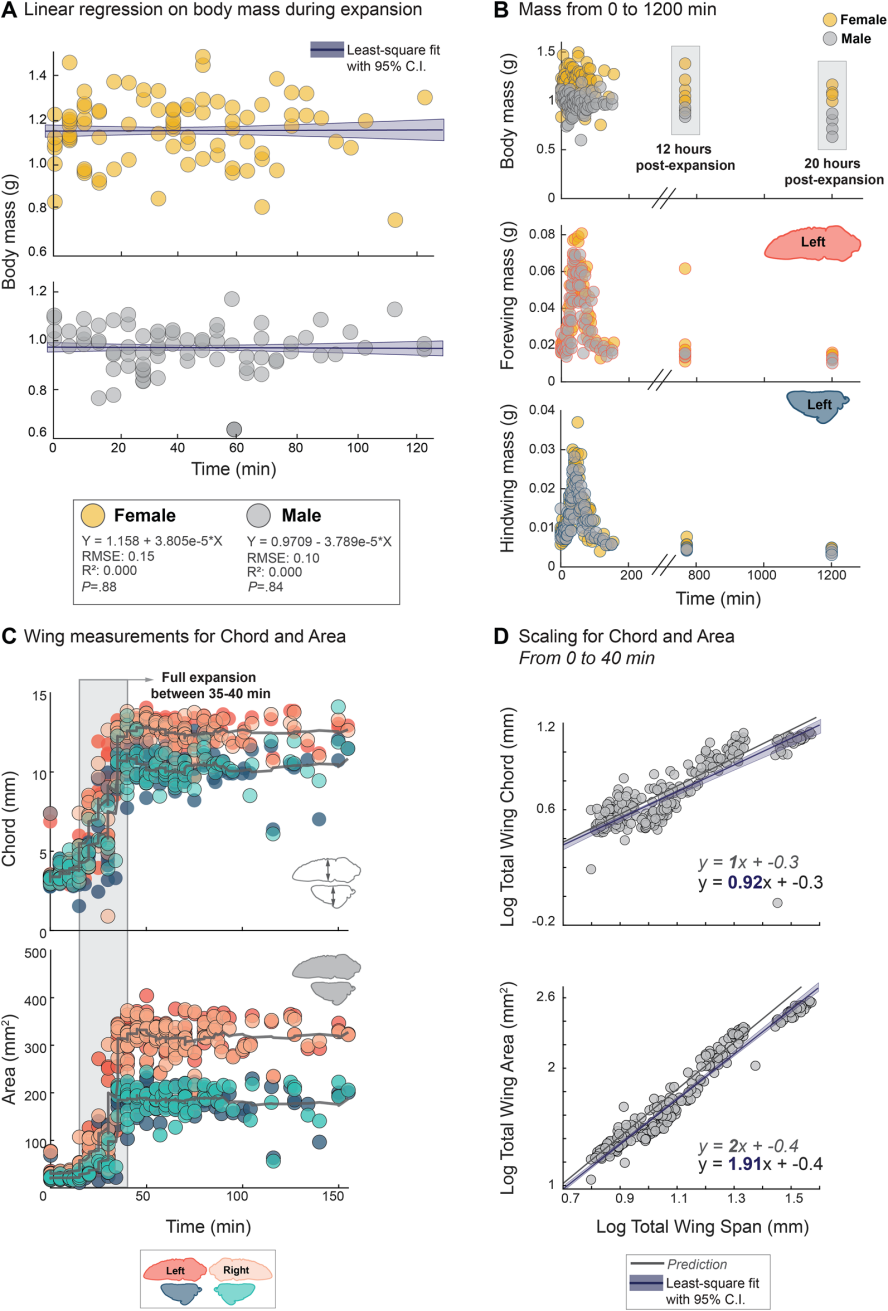
Body mass, area, and chord relationships. (**A**) Body mass trends for female (yellow) and male (gray) cicadas from t = 0 to 120 min. Data were pooled in the main study as there were no effects of sex on the statistical models. (**B**) Masses for body, left forewings, and left hindwings, for female (yellow) and male (gray) cicadas. Only the left side is represented, as there were no significant differences between the left and right wing mass trends. (**C**) Area and chord represented for all wings, both left and right; however, there were no significant differences between left and right measurements. (**D**) Scaling patterns of log total wing chord and area relative to log of wing span. These relationships followed predictions of geometric isometry.

During expansion, span, chord, and area increased until reaching their maximum size (as compared with adult wings) at t = 40 min, after which these values plateaued (Fig. 1C, ii; See Fig. 3 for chord and area). However, wing mass changed both before and after expansion, first increasing to a maximum of 6× initial mass (t = 40 min) and then decreasing to values less than those of the folded wing pad (t = 150 min). Relative to starting wing pad mass, the final adult wings exhibited an overall 27% decrease in mass of the forewings and 51% decrease in the hindwings (*P* < 0.05). Although female cicadas were larger than males, their wing expansion patterns were nearly identical (Fig. 1C,ii–iv). Body mass had no significant effect on wing expansion. Although female cicadas were significantly heavier than male cicadas, there were no statistically significant effects on increasing or decreasing wing mass (Fig. 3A,B). Wing chord and area showed similar trends to wing span (Fig. 3C), and log–log data followed predicted trends expected with scaling by geometric isometry (Fig. 3D).

A scaling analysis of expansion showed that wing span, chord, and area increased with geometric isometry, but mass changed with a large negative allometry (Fig. 1E; see Fig. 3 for chord and area). These results reflect that wing unfurling and inflating are processes in which soft tissue expands primarily in two dimensions, effectively expanding as a flat plate. How expansion is mechanically constrained to two dimensions is unknown, but this process should depend on the material properties of the tissues and their response to hydraulic pressures produced by known mechanisms of flow production: pumping of thoracic wing hearts, flight muscle contractions, and abdominal ventilation^16–18^, controlled by known hormonal cues^7^.

## Discussion

Our data demonstrate that once the cicada wing’s final form is attained, its mass drops precipitously. What mechanisms are responsible for this rapid change? Because a cicada’s cuticle is in the process of sclerotizing and hardening during the first 2 h of wing expansion after eclosion (a process that continues over the next 24–48 h), transcuticular water loss may be high. However, there was no congruent pattern of mass loss in the body (Fig. 1C,iv) whose cuticle was subject to the same ambient environment as the wings. More likely, hemolymph is transported out of the wings by reversing flow using the same mechanisms that pumped it in. Removal of fluid would present a structural challenge to the wings, as large negative pressures might induce collapse of the veins. Because no collapse occurs, the net hydraulic transfer of hemolymph out of the veins should be accompanied by an equivalent volume displacement, possibly by expansion of the tracheal tubes, which has been observed in lepidopterans^12^.

Much of our understanding of wing expansion and the role of hemolymph, pressure, and pumping coordination come from work with the giant moth *Attacus atlas*^9,25^. Using freeze-fixing methods to observe the ratio of hemolymph to tracheae, wing veins were shown to exhibit a dramatic increase in the tracheal fraction from 2 h after wing inflation to 24 h after eclosion. This increase in tracheal volume is tied to the volume change of post-ecdysial diuresis, where water loss changes not only hemolymph viscosity but also internal pressure, driving the wing layers to approach, finish sealing, and fully sclerotize^25^. Other key work on ecdysis involved the desert locust *Schistocerca gregaria*^16^, a species that also relies on gut inflation and expansion of air sacs in the abdomen and thorax for successful wing expansion^26^. If this feature is common among insects, the recruitment of tracheal tubes within the veins would mean that respiratory ventilation also plays a major role in wing development in cicadas.

The role of accessory pumping organs during wing expansion, specifically those thoracic wing hearts that drive hemolymph flow out of the adult wing^1,10^, remains mysterious^9^. In some lepidoptera, cutting off the thoracic pulsatile organs from connection to the wing (through clamping) has no visible effects on wing expansion^25^. However, if thoracic wing hearts are knocked out in *Drosophila*, the wings stay cloudy and do not fully mature^2^. To further explore the role of accessory pumping organs, future experiments could clamp the trailing edge veins (specifically the auxillary cord, which leads back to thoracic pulsatile organs) to determine if wing expansion is still successful^4^.

These data provide a basis for a new understanding of wing expansion, one that should be tested far more broadly in winged insects, which comprise the vast majority of insect species. Future experiments could investigate mass changes in the wing and body^3^, as well as to identify the exact mechanisms that drive the flow and allow the wing to maintain structural integrity (and to not collapse) upon withdrawal of the fluid. Further focus could include thoracic wing heart timing, flight muscle contractions, and abdominal movements, all of which are involved in wing expansion to varying temporal degrees. These factors have been noted previously, but their relative role needs to be quantified.

Despite different modes of wing development (hemi-/holometabolous) across insect species, adult wings likely share a common mechanism of expansion by hydraulic pumping^1,8^. Because a wing must be flexible, light, and strong to allow dynamic behaviors such as flying, mating, and feeding, the transport of hemolymph in and out of a wing during development likely represents a critical process in insect ecology. Furthermore, reducing wing mass after expansion must also decrease wing inertia, since distributed wing mass along the span and chord are major mechanical factors for flapping-wing flyers^22^. The diversity and range of insect wing venation patterns also makes wing expansion an ideal yet understudied candidate for informing new bio-inspired microfluidic devices and soft, deployable robotics^3^. Overall, our results on wing expansion in cicadas provide new understanding of the role of the circulatory system as a major physical mechanism in insect development.

## Methods

### Periodical cicadas

Depending on the brood species and location, periodical cicada nymphs emerge in the hundreds to millions every 13 or 17 years, digging out of the ground and crawling up vertical surfaces to begin the molt to the adult stage and wing expansion. In 2021, Brood X cicadas emerged in the millions. Their high local abundance (densities up to 1000 cicadas/m^2^)^22^ enabled us to collect a large number of samples in a short amount of time, particularly in comparison to annual cicada species, which occur in much lower densities. Brood X contained three species in 2021: *Magicicada septendecim, M. septendecula*, and *M. cassini*. We collected primarily *Magicicada septendecim*, though we also encountered some *M. septendecula* and *M. cassini*. We keyed out the species based on morphological differences^27^. Specifically, *M. septendecim* lacked the clear orange bands present in *M. septendecula* and *M. cassini*, and in our collection, *M. septendecim* was found in large quantities in our area. We rarely found the other two species during our experiment.

### Nymph collection

Active nymphs crawling towards trees were collected by hand and identified at dusk (between 6:30 and 7:30 pm) in Alexandria, VA, USA, from May 15–19, 2021. Nymphs were transported at ambient temperatures (16–26 °C) in coolers to a local residence (transport time, ~10 min) and immediately processed for expansion experiments. Nymphs were allowed to freely climb a vertical netting (mosquito netting, 3× 1.2 m) mounted on a wall (Fig. 2) exposed to ambient air. Nymphs typically climbed a short distance (~1–2 m, for ~5–15 min), settled, and then initiated expansion within minutes after settling. No more than 60 min occurred between collection and eclosion; often, the time to eclosion was much less. Starting on May 17th, nymphs promptly appeared in the grass at dusk, and collection by hand was rapid. As individual cicadas began molting, a timer was started, with time zero demarcated by the emergence of all four of the cicada's wings from the wing pad, out of the exuvia. Labels were placed next to molting cicadas to identify each specimen. Cicadas were taken off the netting at 5-min intervals during wing expansion, placed in a labeled container in the freezer, and quickly processed within 10 min. Freezing of cicadas functioned both to anesthetize them and to pause wing expansion. However, some expansion likely occurred while the insect was cooling.

### Weighing and imaging

During dissection, cicadas were first weighed on a scale (AL104 Analytical Balance, Mettler Toledo; resolution, 0.1 mg), and full-body weights were collected. Each of their wings (left/right forewings and hindwings) were then carefully removed by cutting with scissors (Fine Science Tools, spring scissors) and individually weighed. The left forewing was removed first and weighed, followed by the left hindwing, right forewing, and right hindwing. After dissection, cicadas were quickly wrapped in facial tissues (Kleenex), labeled, and placed back in the freezer. Wrapping cicadas in tissue allowed us to anesthetize them rapidly, limit hemolymph bleeding, damage that the cicada might do to itself, and allowed us to quickly label the cicada (at this point, cicadas could still crawl away unless gently restrained). Wings were arranged on black cardstock and photographed using a DSLR camera (Nikon D850) and macro lens (Sigma 180 mm), using a micro SD card (length, 14.99 mm) as a scale bar. Imaging was illuminated with LED lighting, which was chosen because it does not locally heat the air (or wings).

### Analysis

A total of 800 wings from 200 cicadas were sampled in 5-min intervals, from zero min (wings freed from casing) to 2 h later. Of those specimens, 100 female and 84 male *Magicicada* cicadas were analyzed; wing mass, span (maximum wing length), chord (mid-span wing width), and area were then measured using FIJI software^28^ and analyzed using MATLAB software^29^. Pixel distance was calibrated using the known reference (micro SD card) in each image. Linear distances (wing length and width) were measured manually using the line tool in FIJI. Span was measured as the greatest distance from wing base to wing tip. Wing chord was measured orthogonally to the span as the width of the wing at the mid-span location (i.e., halfway along the span). Area was measured using the free-hand tool to trace the perimeter of the wing, from which wing area was calculated. The 16 male cicadas that are missing from the dataset were removed post-hoc because we deemed them as preliminary: they were collected on the first day of work (May 15th, 2021), prior to refining and standardizing our sampling techniques. Excess specimens that emerged into adult cicadas were released at the site of collection.

### Statistical analysis

A mixed-model ANOVA was used to investigate the effects of time (0 to 120 min with bin size of 5-min increments), sex (male/female), side (left/right), type (forewing/hindwing), and whole-body mass, on wing mass, wing span, wing chord, and wing area. Through preliminary observations on the first day (May 15th), we noted that wings reached full size within an hour, and mass changes stabilized within 2 h, forming the basis of our sampling protocol. All two-way and three-way interactions were included in the initial models. Starting with the three-way interactions, non-significant interactions with the highest p-values, or significant interactions deemed to be of no practical importance, were removed one at a time to obtain a parsimonious final model (Supp. Table 1, Statistics Table). No three-way or two-way interactions were significant and of practical importance; thus, all final models only included main effects. Pairwise comparisons between time points were performed using Tukey’s HSD test. Statistical analyses were performed using JMP Pro 12 (SAS Institute Inc., Cary, NC) with a significance level of 0.05.

## Supplementary Information

Supplementary Information 1.

Supplementary Video 1.

Supplementary Legends.

## Data Availability

Complete tables of cicada wing masses, span, chord, and area and associated summary statistics are freely available at the Socha Lab Github site. Link: https://github.com/TheSochaLab/Transient-use-of-hemolymph-for-hydraulic-wing-expansion-in-cicadas.
