# Supplementary material for: Transient use of hemolymph for hydraulic wing expansion in cicadas
Source: Sci Rep. 2023 Apr 18;13:6298. doi: 10.1038/s41598-023-32533-4 (PMC10113369; doi:10.1038/s41598-023-32533-4)
Supplement: Supplementary file 1 — Supplementary Information 1. [file 41598_2023_32533_MOESM1_ESM.pdf]

Supplemental Information for:

Transient use of hemolymph for hydraulic wing expansion in cicadas

Mary K. Salcedo[1*], Tyler E. Ellis[1], Ángela S. Sáenz[2], Joyce Lu[1], Terrell Worrell[1], Michael L. Madigan[3], and John J. Socha[1]

1 - Biomedical Engineering and Mechanics, Virginia Tech, Blacksburg, VA
2 - Entomology, University of Maryland, College Park, MD
3 – Industrial and Systems Engineering, Virginia Tech, Blacksburg, VA
* To whom correspondence should be addressed. Email: msalcedo@vt.edu

Acknowledgments
A special thanks goes to the University of Maryland Cicada Crew and Steve Young, who contributed thoughtful discussion on cicadas, citizen science observations, and nymphs to the experiment. The authors thank the Parks Service of Alexandria for their thoughtful comments on our project during our stay. The authors thank their fellow Virginia Tech Socha lab mates who joined in the collection process: Joshua Pulliam, Jeff Anderson Jr, Talia Weiss, Noah Goldfarb, and Giovanni Morris. A special thanks goes to Michelle Graham for suggesting the wall of insect mesh to allow cicadas to free-climb. We also thank Alison Henry for the helpful discussion.

Contributions
MKS and JJS designed the experiments. ASS, resident cicada expert, led collection of cicada nymphs. MKS, TEE, TW, and JL timed, dissected, and recorded wing data. MKS, TEE, JL, ASS analyzed the data and made figures. MKS, JJS, and MLM performed the statistical analyses. All co-authors contributed equally to the writing of the manuscript.

Data availability
Complete tables of cicada wing masses, span, chord, and area and associated summary statistics are freely available at the Socha Lab Github site. Link: https://github.com/TheSochaLab/Transient-use-of-hemolymph-for-hydraulic-wing-expansion-in-cicadas

Additional Discussion

Developing pressure in the body and wings

Wing expansion requires a suite of hormonal triggers and motor programs (Carlson 1977, Hughes 1980, I-IV) to ensure normal emergence from the exuviae (juvenile casing) of the head and all appendages, and adequate internal pressure into the wing (Truman 2005). Upon ecdysis, for hemimetabolous insects, the wing is pulled out of its wing pad (protective casing) and hemolymph is transported into the wing (Hughes 1980I, Elliott 1981). Vigorous ventilatory abdominal pumping and wing flexing suggests that inflation with hemolymph is a primary driver of wing expansion (Glaser and Vincent 1979), but air may also play a role. For winged insects, post-eclosion, the process of unfolding and expanding the wings is a critical bottleneck in achieving sexual maturity.

Much of our current understanding of the pressurization mechanisms behind wing expansion have been investigated in species within Orthoptera (locusts, grasshoppers, and crickets). Because expansion behavior is somewhat stereotyped, behavioral events for these orthopterans and other hemipterans tend to follow six main events laid out by Elliott (1981) and Hughes (1980, I), including: 1) Nymph ceases to feed, new adult cuticle is laid down; 2) Nymph selects site for emergence, slowly inflates gut; 3) Gut inflation occurs, old cuticle is split, head, thorax, legs emerge; 4) Metathoracic legs fold; 5) Wings expand; and 6) New cuticle strengthens, gut deflates.

Prior to phase 1, the cuticle begins to soften, likely triggered by bursicon, an important hormone in both controlling and initiating ecdysis (Elliott 1981, White and Ewer 2014). As the head emerges, followed by antennae and legs, old tracheal lining is pulled out of the thoracic and abdominal spiracles. Maximum pressures occur during the process of wriggling and emerging from the juvenile form (Elliott 1981), and waves of muscular contractions are necessary for splitting the cuticle of the exuviae. The insect also extracts itself from its exuvia using deep inspirations into the tracheal system, which occurs in 20-60 second bouts (Elliott 1981). The insect curves backwards, head first, gulping in air to inflate its gut. This causes internal pressure to be uniformly pushed outwards on the exoskeleton, stretching it as it undergoes changing stiffness. The highest pressures occur during phase 3, and steadily decline into phase 5 (Elliott 1981). This process lasts from 15–20 min, depending on the insect species. Hanging from the tip of the exuviae for several minutes (depending on insect size), the nymph flips forward and begins wing expansion. During phase 5 (wing expansion), compression bouts of the expansional motor program result in a pulling of the wings laterally (Hughes 1980, I-III), which appears as a strong flex of the flight muscles.

Developing the necessary pressures within the insect body to extract itself from its juvenile form, to inflate its entire exoskeleton, and to push hemolymph into the wings, requires a complex sequence of timed events. Eclosion can fail at any point (White *et al*., 1979), resulting in the insect's head and/or appendages remaining within exuviae (White and Ewer 2014), causing wing expansion to completely cease. Additionally, if the wing hinges are damaged and leaking hemolymph, the wing fails to extend to its full length (personal observation, MKS). In the last

stages of wing expansion, specifically for Orthoptera, if multiple appendages have been lost, the insect may not be able to fold its newly expanded adult wings into their proper adult configuration.

Despite this knowledge, overall our understanding of wing expansion and the role of circulation across insect phylogeny is piecemeal. Circulation in insect wings was described by Arnold (1964) across 14 insect orders and 100 species, but without connection to wing structure and physiology (Salcedo and Socha 2020). Physiology of thoracic wing hearts was described across holometabolous and hemimetablous insects, but quantitative measurement of hemolymph flows within the wing or relation to wing expansion is lacking (Krenn and Pass, 1994, 1995). Lastly, previous studies of ecdysis have measured muscular coordination and behavior, without connection to the changing wing pressures, structure, or mass changes. Overall, our data on cicadas broaden our understanding of wing expansion, quantifying a feature of wing expansion (mass change in the wings) that has not previously been understood.

Why cicadas? An ecological perspective

Investigating non-model systems like cicadas can lend insight into broader issues such as climate change. Periodical cicadas lay eggs in tree branches; these eggs hatch, and the nymphs drop to the ground, where they feed on root xylem for the next 13-17 years. With increased urbanization, habitat loss causes a population decrease. It is already clear that higher temperatures are causing brood extinction (White *et al.*, 1979, Cooley *et al.*, 2013), and with climate change, effects on insect development, such as early emergence, are already seen in periodical cicadas under warmer conditions (Heath 1968, Moriyama and Numata, 2019). At our experiment's locale, cicadas largely emerged from trees lining the street, where pavement warmth and daylight exposure cued cicadas to emerge 2-3 weeks before those in forested areas. For periodical cicadas, synchronicity is a leading survival mechanism known as 'predator satiation' (Koenig and Liebhold, 2013), whereby the emergence of individuals at different intervals of time could weaken their advantage in numbers.

SPAN	Nparm	DF	DF Den	F Ratio	Prob > F	Note
Sex	1	1	138.7	0.9488	0.3317	
Time (min)	24	24	140.2	102.5122	<.0001	
Whole mass (g)	1	1	148.3	0.4828	0.4882	
Wing mass (g)	1	1	571.2	221.8368	<.0001	
Wing side (L/R)	1	1	445	0.0421	0.8374	
Wing type (Fore/hind)	1	1	559.6	750.2265	<.0001	
Time (min)*Wing type (Fore/hind)	24	24	448	63.2596	<.0001	Interaction
CHORD	Nparm	DF	DF Den	F Ratio	Prob > F	
Sex	1	1	127.6	2.1668	0.1435	
Time (min)	24	24	128	76.4007	<.0001	
Whole mass (g)	1	1	134	4.8148	0.0299	
Wing mass (g)	1	1	554.3	100.1525	<.0001	
Wing side (L/R)	1	1	446.9	0.0771	0.7814	
Wing type (Fore/hind)	1	1	526.8	12.72	<.0001	
AREA	Nparm	DF	DF Den	F Ratio	Prob > F	
Sex	1	1	129.3	2.6177	0.1081	
Time (min)	24	24	130.8	76.2139	<.0001	
Whole mass (g)	1	1	135.9	4.283	0.0404	
Wing mass (g)	1	1	524.8	170.8598	<.0001	
Wing side (L/R)	1	1	418.6	1.5096	0.2199	
Wing type (Fore/hind)	1	1	512	356.7712	<.0001	
Time (min)*Wing type (Fore/hind)	24	24	421	69.7361	<.0001	Interaction
WING MASS	Nparm	DF	DF Den	F Ratio	Prob > F	
Sex	1	1	142.1	4.4040	0.0376	
Time (min)	24	24	141.4	37.9776	<.0001	
Whole mass (g)	1	1	141.9	36.1532	<.0001	
Wing side (L/R)	1	1	500.5	17.6235	<.0001	
Wing type (Fore/hind)	1	1	500.9	8120.234	<.0001	

Supp. Table. 1. Statistics from mixed-effects model testing four dependent variables: span, chord, area, and mass. *Terms: Nparm - number of parameters, DF - degrees of freedom, DF Den - degrees of freedom density, F Ratio, Prob > F - P Value*

References (Alphabetical)

1. Arnold, J.W., 1964. Blood circulation in insect wings. *The Memoirs of the Entomological Society of Canada*, *96*(S38), pp.5-60.
2. Carlson, J.R., 1977. The imaginal ecdysis of the cricket (*Teleogryllus oceanicus*). *Journal of Comparative Physiology*, *115*(3), pp.299-317.
3. Cooley, J.R., Marshall, D.C., Simon, C., Neckermann, M.L. and Bunker, G., 2013. At the limits: Habitat suitability modeling of northern 17−year periodical cicada extinctions (Hemiptera: *Magicicada spp.)*. *Global Ecology and Biogeography*, *22*(4), pp.410-421.
4. Elliott, C.J.H., 1981. The expansion of *Schistocerca gregaria* at the imaginal ecdysis: The mechanical properties of the cuticle and the internal pressure. *Journal of Insect Physiology*, *27*(10), pp.695-704.
5. Glaser, A.E. and Vincent, J.F.V., 1979. The autonomous inflation of insect wings. *Journal of*

Insect Physiology, *25*(4), pp.315-318.
6. Heath, J.E., 1968. Thermal synchronization of emergence in periodical "17-year" cicadas (Homoptera, Cicadidae, Magicicada). *American Midland Naturalist*, pp.440-448.
7. Hughes, T.D., 1980. The imaginal ecdysis of the desert locust, *Schistocerca gregaria*. I. A description of the behaviour. *Physiological Entomology*, *5*(1), pp.47-54.
8. Hughes, T.D., 1980. The imaginal ecdysis of the desert locust, *Schistocerca gregaria*. II. Motor activity underlying the pre−emergence and emergence behaviour. *Physiological Entomology*, *5*(1), pp.55-71.
9. Hughes, T.D., 1980. The imaginal ecdysis of the desert locust, *Schistocerca gregaria*. III. Motor activity underlying the expansional and post−expansional behaviour. *Physiological Entomology*, *5*(2), pp.141-152.
10. Hughes, T.D., 1980. The imaginal ecdysis of the desert locust, *Schistocerca gregaria.* IV. The role of the gut. *Physiological Entomology*, *5*(2), pp.153-164.
11. Moriyama, M. and Numata, H., 2019. Ecophysiological responses to climate change in cicadas. *Physiological Entomology*, *44*(2), pp.65-76.
12. Koenig, W.D. and Liebhold, A.M., 2013. Avian predation pressure as a potential driver of periodical cicada cycle length. *The American Naturalist*, *181*(1), pp.145-149.
13. Krenn, H.W. and Pass, G., 1995. Morphological diversity and phylogenetic analysis of wing circulatory organs in insects, part II. Holometabola. *Zoology*, *98*, pp.147-147.
14. Krenn, H.W. and Pass, G., 1994. Morphological diversity and phylogenetic analysis of wing circulatory organs in insects, part I: Non-Holometabola. *Zoology*, *98*(1), pp.7-22.
15. Salcedo, M.K. and Socha, J.J., 2020. Circulation in insect wings. *Integrative and Comparative Biology*, *60*(5), pp.1208-1220.
16. Truman, J.W., 2005. Hormonal control of insect ecdysis: Endocrine cascades for coordinating behavior with physiology. *Vitamins & Hormones*, *73*, pp.1-30.
17. White, J., Lloyd, M. and Zar, J.H., 1979. Faulty eclosion in crowded suburban periodical cicadas: populations out of control. *Ecology*, *60*(2), pp.305-315.
