# Supplementary material for: Transient use of hemolymph for hydraulic wing expansion in cicadas
Source: Sci Rep. 2023 Apr 18;13:6298. doi: 10.1038/s41598-023-32533-4 (PMC10113369; doi:10.1038/s41598-023-32533-4)
Supplement: Supplementary file 3 — Supplementary Legends. [file 41598_2023_32533_MOESM3_ESM.docx]

**Supplement Video. Time lapse of eclosion and wing expansion in cicadas.** A time lapse recording of a *Magicicada* species emerging after dusk. At the start of this video, the dorsal thorax is split and the adult is wriggling out. Once the folded wings are clear of the wing pad structures on its back, we considered it “time zero” for sampling purposes. Eclosion and the immediate wing expansion takes approximately 40 min. During the backbend phase, old tracheae linings are clearly seen being pulled from the adult old tracheal linings. (Temperature, 13 degrees Celsius; Time lapse, 1 photo per 10 sec; Time lapse duration, 37 sec; camera, CanonEOS Rebel T7i; playback frame rate, 29.97 fps)
